# Crystal structure of di­methyl­formamidium bis­(tri­fluoro­methane­sulfon­yl)amide: an ionic liquid

**DOI:** 10.1107/S2056989016012251

**Published:** 2016-08-12

**Authors:** Allan Jay P. Cardenas, Molly O’Hagan

**Affiliations:** a Pacific Northwest National Laboratory, PO Box 999 MSIN: K2-57, Richland, WA 99352, USA

**Keywords:** crystal structure, ionic liquid, electrolyte, hydrogen bond

## Abstract

At 100 K, the title mol­ecular salt, C_3_H_8_NO^+^·C_2_F_6_NO_4_S_2_^−^, has ortho­rhom­bic (*P*2_1_2_1_2_1_) symmetry; the amino H atom of bis­(tri­fluoro­methane­sulfon­yl)amine (HNTf_2_) was transferred to the basic O atom of di­methyl­formamide (DMF) when the ionic liquid components were mixed. The structure displays an O—H⋯N hydrogen bond, which links the cation to the anion, which is reinforced by a non-conventional C—H⋯O inter­action, generating an *R*_2_^2^(7) loop. A further very weak C—H⋯O inter­action generates an [001] chain.

## Abstract 2

The cation and anion of the title salt are linked by an O—H⋯N hydrogen bond and a C—H⋯O inter­action, resulting in a high viscosity and a crystallization temperature slightly lower than ambient temperature.

## Chemical context   

A ionic liquid, also known as a liquid electrolyte, is a salt or an ion pair that remains in a liquid state below 373 K (Ghandi, 2014 ▸): such species extend the selection of solvents or media of chemical processes. The study of its solid-state structure can facilitate the exploration of other inter­molecular forces of attraction besides electrostatic forces that govern the properties of these ionic liquids such as melting point, acidity, ion mobility, diffusion and viscosity. In this study we report the crystal structure of an organic liquid salt formed by a proton-transfer reaction between bis­(tri­fluoro­methane­sulfon­yl)amine and di­methyl­formamide. This protic ionic liquid has been used as a solvent, an electrolyte and a substrate for electrocatalysis (Hou *et al.*, 2014 ▸).
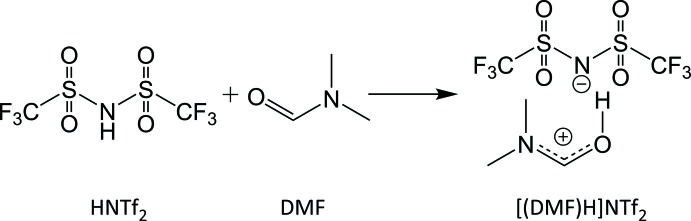


## Structural commentary   

**Figure 1:**
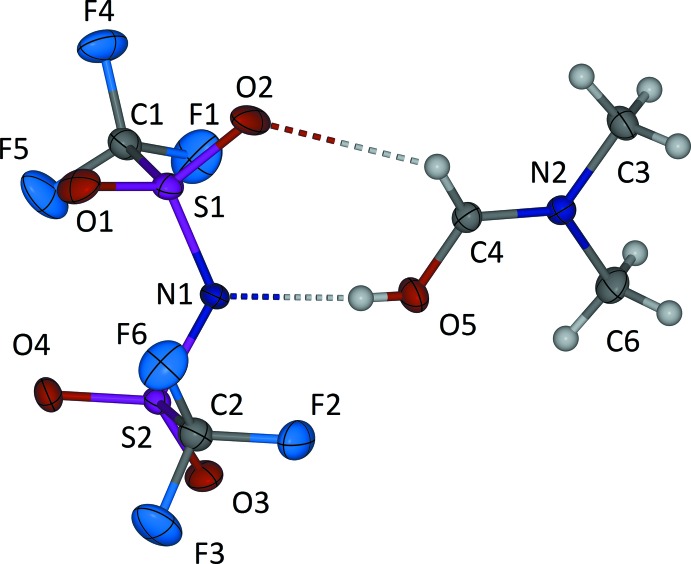
The mol­ecular structure of the title compound, showing displacement ellipsoids drawn at the 50% probability level. Hydrogen bonds are shown as dashed lines.

The asymmetric unit consists of one bis­(tri­fluoro­methane­sulfon­yl)amide anion and one di­methyl­formamidium cation (Fig. 1 ▸): when the components were mixed, the acidic N—H proton of HNTf_2_ was transfered to the formyl group of di­methyl­formamide. The di­methyl­formamidium C4—O5 and N2—C4 bond lengths are 1.2983 (16) and 1.2888 (15) Å respectively, which reflect the delocalization of charge *via* π-electrons. The N2—C4—O5 angle does not deviate from the expected 120° of an *sp* ^2^-hybridized carbon atom [120.37 (11)°]. The bis­(tri­fluoro­methane­sulfon­yl)amide anion features S1—N1 and S2—N1 bond distances of 1.6035 (11) and 1.5947 (11) Å, respectively.

## Supra­molecular features   

The ion pair features two hydrogen bonds (Table 1 ▸). One is between the acidic hydrogen atom attached to the formyl oxygen atom of the di­methyl­formamidium cation and the nitro­gen atom of the bis­(tri­fluoro­methane­sulfon­yl)amide anion: the H⋯N distance is 1.98 (3) Å. The other is a non-conventional C—H⋯O hydrogen bond between the formyl hydrogen atom of the di­methyl­formamidium cation and one of the sulfoxide oxygen atoms of the anion (Desiraju, 1991 ▸). The C4—H⋯O2 distance is 2.57 Å (Table 1 ▸). Together, these generate an 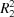
(7) loop. A further very weak C—H⋯O inter­action links the ion pairs into an [001] chain.

## Database survey   

A CSD search (Web CSD version 1.1.1; May 4, 2016) found no structures that have the same ion pairing. Some structures feature the same bis­(tri­fluoro­methane­sulfon­yl)amide anion but different cations, which are usually metal complexes.

## Synthesis and crystallization   

A literature procedure was followed to synthesize [(DMF)H]NTf_2_ (I) (Hou *et al.*, 2014 ▸). Equimolar amounts of of di­methyl­formamide (17.6 mmol, 1.29 g) and bis­(tri­fluoro­methane­sulfon­yl)amine (17.8 mmol, 5.0g) were mixed tog­ether after cooling each reagent to 238 K. The solution was stirred at room temperature until it formed a light-yellow viscous solution. The solution was then left to stand undisturbed at room temperature and colorless blocks of (I) were isolated.

## Refinement   

Crystal data, data collection and structure refinement details are summarized in Table 2 ▸. H atoms were positioned geometrically and allowed to ride on their parent atoms: C—H = 0.93–0.96 Å with *U*_iso_(H) = 1.5*U*_eq_(C-meth­yl) and 1.2*U*_eq_(C) for other H atoms. The methyl groups were refined as rotating groups.

## Supplementary Material

Crystal structure: contains datablock(s) I. DOI: 10.1107/S2056989016012251/hb7586sup1.cif

Structure factors: contains datablock(s) I. DOI: 10.1107/S2056989016012251/hb7586Isup2.hkl

Supporting information file. DOI: 10.1107/S2056989016012251/hb7586Isup3.cml

CCDC reference: 1496417

Additional supporting information: crystallographic information; 3D view; checkCIF report

## Figures and Tables

**Table 1:** Hydrogen-bond geometry (Å, °)

*D*—H⋯*A*	*D*—H	H⋯*A*	*D*⋯*A*	*D*—H⋯*A*
O5—H5⋯N1	0.74 (3)	1.98 (3)	2.7139 (14)	172 (2)
C4—H4⋯O2	0.93	2.57	3.2694 (16)	132
C4—H4⋯O4^i^	0.93	2.63	3.4773 (16)	152

Symmetry code: (i) 
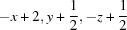
.

**Table 2:** Experimental details

Crystal data	
Chemical formula	C_3_H_8_NO^+^·C_2_F_6_NO_4_S_2_^−^
*M*_r_	354.25
Crystal system, space group	Orthorhombic, *P*2_1_2_1_2_1_
Temperature (K)	100
*a*, *b*, *c* (Å)	9.0254 (9), 11.4601 (12), 12.3621 (14)
*V* (Å^3^)	1278.6 (2)
*Z*	4
Radiation type	Mo *K*α
μ (mm^−1^)	0.51
Crystal size (mm)	0.40 × 0.30 × 0.30

Data collection	
Diffractometer	Bruker APEXII CCD
Absorption correction	Multi-scan (*SADABS*; Bruker, 2015 ▸)
*T*_min_, *T*_max_	0.582, 0.746
No. of measured, independent and observed [*I* > 2σ(*I*)] reflections	15926, 4274, 3989
*R*_int_	0.033
(sin θ/λ)_max_ (Å^−1^)	0.739

Refinement	
*R*[*F* ^2^ > 2σ(*F* ^2^)], *wR*(*F* ^2^), *S*	0.025, 0.066, 1.06
No. of reflections	4274
No. of parameters	187
H-atom treatment	H-atom parameters constrained
Δρ_max_, Δρ_min_ (e Å^−3^)	0.29, −0.30
Absolute structure	Flack (1983 ▸)
Absolute structure parameter	−0.01 (4)

Computer programs: *APEX2* and *SAINT* (Bruker, 2013 ▸), *SHELXS97* (Sheldrick, 2008 ▸), *SHELXL2014* (Sheldrick, 2015 ▸) and *OLEX2* (Dolomanov *et al.*, 2009 ▸).

## supplementary crystallographic information

### Crystal data

**Table:**

C_3_H_8_NO^+^·C_2_F_6_NO_4_S_2_^−^	*D*_x_ = 1.840 Mg m^−^^3^
*M**_r_* = 354.25	Mo *K*α radiation, λ = 0.71073 Å
Orthorhombic, *P*2_1_2_1_2_1_	Cell parameters from 9937 reflections
*a* = 9.0254 (9) Å	θ = 2.4–31.5°
*b* = 11.4601 (12) Å	µ = 0.51 mm^−^^1^
*c* = 12.3621 (14) Å	*T* = 100 K
*V* = 1278.6 (2) Å^3^	Block, colourless
*Z* = 4	0.40 × 0.30 × 0.30 mm
*F*(000) = 712	

### Data collection

**Table:**

Bruker APEXII CCD diffractometer	4274 independent reflections
Radiation source: fine-focus sealed tube	3989 reflections with *I* > 2σ(*I*)
Graphite monochromator	*R*_int_ = 0.033
φ and ω scans	θ_max_ = 31.7°, θ_min_ = 2.4°
Absorption correction: multi-scan (SADABS; Bruker, 2015)	*h* = −13→12
*T*_min_ = 0.582, *T*_max_ = 0.746	*k* = −16→16
15926 measured reflections	*l* = −17→18

### Refinement

**Table:**

Refinement on *F*^2^	Secondary atom site location: difference Fourier map
Least-squares matrix: full	Hydrogen site location: inferred from neighbouring sites
*R*[*F*^2^ > 2σ(*F*^2^)] = 0.025	H-atom parameters constrained
*wR*(*F*^2^) = 0.066	*w* = 1/[σ^2^(*F*_o_^2^) + (0.0363*P*)^2^] where *P* = (*F*_o_^2^ + 2*F*_c_^2^)/3
*S* = 1.06	(Δ/σ)_max_ = 0.001
4274 reflections	Δρ_max_ = 0.29 e Å^−^^3^
187 parameters	Δρ_min_ = −0.30 e Å^−^^3^
0 restraints	Absolute structure: Flack (1983)
Primary atom site location: structure-invariant direct methods	Absolute structure parameter: −0.01 (4)

### Special details

**Table:**

Experimental. Absorption correction: SADABS-2014/5 (Bruker,2014/5) was used for absorption correction. wR2(int) was 0.0777 before and 0.0530 after correction. The Ratio of minimum to maximum transmission is 0.7795. The λ/2 correction factor is 0.00150.
Geometry. All esds (except the esd in the dihedral angle between two l.s. planes) are estimated using the full covariance matrix. The cell esds are taken into account individually in the estimation of esds in distances, angles and torsion angles; correlations between esds in cell parameters are only used when they are defined by crystal symmetry. An approximate (isotropic) treatment of cell esds is used for estimating esds involving l.s. planes.
Refinement. Refinement of F^2^ against ALL reflections. The weighted R-factor wR and goodness of fit S are based on F^2^, conventional R-factors R are based on F, with F set to zero for negative F^2^. The threshold expression of F^2^ > 2sigma(F^2^) is used only for calculating R-factors(gt) etc. and is not relevant to the choice of reflections for refinement. R-factors based on F^2^ are statistically about twice as large as those based on F, and R- factors based on ALL data will be even larger.

### Fractional atomic coordinates and isotropic or equivalent isotropic displacement parameters (Å^2^)

**Table:**

	*x*	*y*	*z*	*U*_iso_*/*U*_eq_	
S1	0.95481 (3)	0.01794 (3)	0.22331 (2)	0.02018 (7)	
S2	1.05136 (3)	−0.05314 (3)	0.43097 (2)	0.01784 (6)	
C3	1.44513 (14)	0.35694 (12)	0.10155 (11)	0.0239 (2)	
H3A	1.3456	0.3670	0.0757	0.036*	
H3B	1.5095	0.3401	0.0417	0.036*	
H3C	1.4774	0.4272	0.1366	0.036*	
F1	1.14157 (11)	−0.10684 (12)	0.11057 (9)	0.0507 (3)	
F2	1.09712 (11)	0.15500 (8)	0.51280 (8)	0.0352 (2)	
F3	0.97455 (13)	0.03798 (10)	0.61557 (7)	0.0455 (3)	
F4	0.92510 (12)	−0.09329 (9)	0.04146 (7)	0.0428 (3)	
F5	0.96239 (14)	−0.20423 (8)	0.17885 (9)	0.0472 (3)	
F6	0.86942 (10)	0.11845 (9)	0.47659 (8)	0.0413 (2)	
O1	0.80609 (10)	−0.00178 (9)	0.25752 (9)	0.0324 (3)	
O2	0.99064 (13)	0.11860 (9)	0.16080 (8)	0.0338 (3)	
O3	1.19216 (10)	−0.08639 (9)	0.47431 (8)	0.0276 (2)	
O4	0.92949 (10)	−0.13268 (9)	0.43684 (8)	0.0250 (2)	
N1	1.07837 (11)	0.00592 (10)	0.31584 (9)	0.0210 (2)	
N2	1.44952 (11)	0.25990 (9)	0.17894 (8)	0.01757 (19)	
O5	1.33633 (10)	0.11905 (9)	0.27429 (9)	0.0260 (2)	
C1	0.99834 (15)	−0.10436 (13)	0.13368 (11)	0.0246 (3)	
C2	0.99431 (14)	0.07208 (13)	0.51356 (11)	0.0253 (3)	
C6	1.59693 (13)	0.22881 (13)	0.22087 (12)	0.0275 (3)	
H6A	1.5879	0.1646	0.2703	0.041*	
H6B	1.6389	0.2947	0.2578	0.041*	
H6C	1.6602	0.2068	0.1618	0.041*	
C4	1.33073 (12)	0.20433 (11)	0.20528 (10)	0.0193 (2)	
H4	1.2405	0.2254	0.1747	0.023*	
H5	1.265 (3)	0.0923 (19)	0.2902 (16)	0.051 (6)*	

### Atomic displacement parameters (Å^2^)

**Table:**

	*U*^11^	*U*^22^	*U*^33^	*U*^12^	*U*^13^	*U*^23^
S1	0.02305 (12)	0.01838 (15)	0.01912 (13)	0.00063 (11)	−0.00390 (11)	0.00356 (11)
S2	0.01587 (10)	0.01937 (14)	0.01828 (12)	−0.00017 (10)	−0.00079 (10)	0.00504 (11)
C3	0.0222 (5)	0.0239 (7)	0.0257 (6)	−0.0006 (5)	0.0031 (5)	0.0054 (5)
F1	0.0329 (5)	0.0708 (8)	0.0485 (6)	0.0044 (5)	0.0119 (4)	−0.0170 (6)
F2	0.0436 (5)	0.0245 (5)	0.0374 (5)	−0.0066 (4)	−0.0092 (4)	−0.0004 (4)
F3	0.0703 (7)	0.0429 (6)	0.0232 (4)	−0.0029 (6)	0.0124 (5)	0.0005 (4)
F4	0.0657 (7)	0.0352 (5)	0.0276 (4)	−0.0002 (5)	−0.0181 (5)	−0.0046 (4)
F5	0.0837 (8)	0.0191 (4)	0.0389 (5)	−0.0046 (5)	0.0122 (6)	0.0027 (4)
F6	0.0301 (4)	0.0410 (6)	0.0530 (6)	0.0141 (4)	−0.0011 (4)	−0.0103 (5)
O1	0.0185 (4)	0.0475 (7)	0.0310 (5)	0.0074 (4)	−0.0046 (4)	0.0018 (5)
O2	0.0586 (7)	0.0185 (5)	0.0244 (5)	−0.0065 (5)	−0.0123 (5)	0.0063 (4)
O3	0.0198 (4)	0.0349 (6)	0.0282 (5)	0.0041 (4)	−0.0056 (4)	0.0067 (4)
O4	0.0234 (4)	0.0233 (5)	0.0282 (5)	−0.0052 (3)	0.0004 (4)	0.0070 (4)
N1	0.0199 (4)	0.0258 (6)	0.0174 (4)	−0.0036 (4)	−0.0020 (4)	0.0069 (4)
N2	0.0162 (4)	0.0197 (5)	0.0167 (4)	0.0011 (4)	0.0015 (4)	−0.0013 (4)
O5	0.0199 (4)	0.0285 (5)	0.0295 (5)	−0.0032 (4)	0.0019 (4)	0.0088 (4)
C1	0.0294 (5)	0.0225 (7)	0.0220 (6)	−0.0014 (5)	0.0003 (5)	0.0024 (5)
C2	0.0275 (5)	0.0252 (7)	0.0231 (6)	−0.0001 (5)	0.0006 (5)	0.0013 (5)
C6	0.0166 (5)	0.0322 (8)	0.0336 (7)	−0.0001 (5)	−0.0030 (5)	0.0034 (6)
C4	0.0172 (4)	0.0223 (6)	0.0184 (5)	−0.0003 (4)	0.0013 (4)	−0.0011 (5)

### Geometric parameters (Å, º)

**Table:**

S1—O1	1.4253 (10)	F2—C2	1.3282 (16)
S1—O2	1.4256 (11)	F3—C2	1.3321 (16)
S1—N1	1.6035 (11)	F4—C1	1.3239 (16)
S1—C1	1.8293 (15)	F5—C1	1.3141 (17)
S2—O3	1.4307 (9)	F6—C2	1.3273 (16)
S2—O4	1.4304 (9)	N2—C6	1.4716 (15)
S2—N1	1.5947 (11)	N2—C4	1.2888 (15)
S2—C2	1.8349 (15)	O5—C4	1.2983 (16)
C3—H3A	0.9600	O5—H5	0.74 (2)
C3—H3B	0.9600	C6—H6A	0.9600
C3—H3C	0.9600	C6—H6B	0.9600
C3—N2	1.4675 (16)	C6—H6C	0.9600
F1—C1	1.3242 (16)	C4—H4	0.9300
			
O1—S1—O2	120.18 (7)	C4—O5—H5	116.8 (16)
O1—S1—N1	115.44 (6)	F1—C1—S1	110.90 (11)
O1—S1—C1	105.10 (7)	F4—C1—S1	109.94 (10)
O2—S1—N1	107.36 (6)	F4—C1—F1	107.68 (12)
O2—S1—C1	104.06 (7)	F5—C1—S1	110.91 (10)
N1—S1—C1	102.53 (6)	F5—C1—F1	108.30 (13)
O3—S2—N1	108.15 (6)	F5—C1—F4	109.03 (13)
O3—S2—C2	104.43 (6)	F2—C2—S2	111.07 (9)
O4—S2—O3	119.62 (6)	F2—C2—F3	108.06 (11)
O4—S2—N1	115.68 (6)	F3—C2—S2	109.56 (10)
O4—S2—C2	104.73 (6)	F6—C2—S2	111.09 (9)
N1—S2—C2	101.98 (6)	F6—C2—F2	107.72 (12)
H3A—C3—H3B	109.5	F6—C2—F3	109.25 (12)
H3A—C3—H3C	109.5	N2—C6—H6A	109.5
H3B—C3—H3C	109.5	N2—C6—H6B	109.5
N2—C3—H3A	109.5	N2—C6—H6C	109.5
N2—C3—H3B	109.5	H6A—C6—H6B	109.5
N2—C3—H3C	109.5	H6A—C6—H6C	109.5
S2—N1—S1	124.53 (6)	H6B—C6—H6C	109.5
C3—N2—C6	115.95 (10)	N2—C4—O5	120.37 (11)
C4—N2—C3	121.11 (11)	N2—C4—H4	119.8
C4—N2—C6	122.92 (11)	O5—C4—H4	119.8
			
C3—N2—C4—O5	−179.64 (11)	O4—S2—N1—S1	21.78 (11)
O1—S1—N1—S2	14.69 (11)	O4—S2—C2—F2	−178.35 (9)
O1—S1—C1—F1	−169.55 (11)	O4—S2—C2—F3	62.34 (10)
O1—S1—C1—F4	71.48 (11)	O4—S2—C2—F6	−58.47 (11)
O1—S1—C1—F5	−49.18 (12)	N1—S1—C1—F1	−48.50 (12)
O2—S1—N1—S2	151.76 (9)	N1—S1—C1—F4	−167.47 (9)
O2—S1—C1—F1	63.26 (12)	N1—S1—C1—F5	71.88 (11)
O2—S1—C1—F4	−55.70 (11)	N1—S2—C2—F2	−57.43 (10)
O2—S1—C1—F5	−176.36 (10)	N1—S2—C2—F3	−176.74 (9)
O3—S2—N1—S1	159.07 (8)	N1—S2—C2—F6	62.45 (11)
O3—S2—C2—F2	55.11 (11)	C1—S1—N1—S2	−98.96 (9)
O3—S2—C2—F3	−64.20 (10)	C2—S2—N1—S1	−91.20 (9)
O3—S2—C2—F6	174.99 (10)	C6—N2—C4—O5	2.30 (19)

### Hydrogen-bond geometry (Å, º)

**Table:**

*D*—H···*A*	*D*—H	H···*A*	*D*···*A*	*D*—H···*A*
O5—H5···N1	0.74 (3)	1.98 (3)	2.7139 (14)	172 (2)
C4—H4···O2	0.93	2.57	3.2694 (16)	132
C4—H4···O4^i^	0.93	2.63	3.4773 (16)	152

Symmetry code: (i) −*x*+2, *y*+1/2, −*z*+1/2.
